# Mechanistic insights into filamentous phage integration in *Vibrio cholerae*

**DOI:** 10.3389/fmicb.2014.00650

**Published:** 2014-11-28

**Authors:** Bhabatosh Das

**Affiliations:** Centre for Human Microbial Ecology, Translational Health Science and Technology InstituteGurgaon, India

**Keywords:** CTXΦ, VGJΦ, TLCΦ, XerC, XerD, *dif*, *attP*

## Abstract

*Vibrio cholerae*, the etiological agent of acute diarrhoeal disease cholera, harbors large numbers of lysogenic filamentous phages, contribute significantly to the host pathogenesis and provide fitness factors to the pathogen that help the bacterium to survive in natural environment. Most of the vibriophage genomes are not equipped with integrase and thus exploit two host-encoded tyrosine recombinases, XerC and XerD, for lysogenic conversion. Integration is site-specific and it occurs at dimer resolution site (*dif*) of either one or both chromosomes of *V. cholerae*. Each *dif* sequence contains two recombinase-binding sequences flanking a central region. The integration follows a sequential strand exchanges between *dif* and *attP* sites within a DNA-protein complex consisting of one pair of each recombinase and two DNA fragments. During entire process of recombination, both the DNA components and recombinases of the synaptic complex keep transiently interconnected. Within the context of synaptic complex, both of the actuated enzymes mediate cleavage of phosphodiester bonds. First cleavage generates a phosphotyrosyl-linked recombinase-DNA complex at the recombinase binding sequence and free 5′-hydroxyl end at the first base of the central region. Following the cleavage, the exposed bases with 5′-hydroxyl ends of the central region of *dif* and *attP* sites melt from their complementary strands and react with the recombinase-DNA phosphotyrosyl linkage of their recombining partner. Subsequent ligation between *dif* and *attP* strands requires complementary base pair interactions at the site of phosphodiester bond formation. Integration mechanism is mostly influenced by the compatibility of *dif* and *attP* sequences. *dif* sites are highly conserved across bacterial phyla. Different phage genomes have different *attP* sequences; therefore they rely on different mechanisms for integration. Here, I review our current understanding of integration mechanisms used by the vibriophages.

## Introduction

Bacterial pathogens evolve continuously to adapt to the changing environment by adopting multiple mobile genetic elements into their compact, modularly organized mosaic genomes to help combat the environmental factors that are detrimental to their subsistence (Frost et al., 2005; Mercier et al., 2008; Hassan et al., 2010). *Vibrio cholerae*, the noxious enteric pathogen with extraordinary fitness competence resides in multiple niches across continents. A variety of the vibrio strains have acquired multiple genetic traits in their genomes (Heidelberg et al., 2000; Sack et al., 2004; Chun et al., 2009), with the purpose of contributing to the toxin production (Waldor and Mekalanos, 1996), intestinal colonization (Rhine and Taylor, 1994), disease development (Herrington et al., 1988), antimicrobial resistance (Mazel and Davies, 1998; Beaber et al., 2004), cell division (Hassan et al., 2010), and survival in aquatic as well as gut environments (Davies et al., 2012). In *V. cholerae*, most of the horizontally acquired genetic traits integrate site-specifically within a short region of sequence identity shared by the host chromosome and the integrative mobile genetic elements (IMGEs), using self- or host-encoded tyrosine recombinases (Huber and Waldor, 2002; Rajanna et al., 2003; Hazen et al., 2010; Das et al., 2013; Banerjee et al., 2014). Integration might be reversible or irreversible, depending upon the structures of pre- and post-integrative attachment sequences (Das et al., 2011a, 2013, 2014). Tyrosine recombinases can bind double stranded as well as folded single stranded DNA of the acquired exogenous genetic elements (Val et al., 2005; Mazel, 2006; Das et al., 2011b). Different acquired genetic traits reported in *V. cholerae* are heterogeneous, and they recognize different receptors for infection (Herrington et al., 1988; Campos et al., 2010; Das et al., 2013), follow different mechanisms to deliver DNA into the host cytoplasm (Heilpern and Waldor, 2000), have a wide range of genomic content (Heidelberg et al., 2000; Faruque and Mekalanos, 2003), and most importantly rely on catalytic activities of different recombinases for chromosomal integration (Rajanna et al., 2003; Das et al., 2010, 2011b; Midonet et al., in press). In a broader sense, these IMGEs can be classified into two different groups, depending upon the presence and absence of recombinases, the enzymes essential for their integration to the host genome. IMGEs, which encode and rely on their own recombinases for integration, are generally large in size and integrates specifically in front of tRNA or tmRNA operon (Karaolis et al., 1999; Heidelberg et al., 2000). On the other hand, IMGEs exploit host-encoded tyrosine recombinases (IMEX), are small in size and integrate at the dimer resolution sites (*dif*) of host chromosomes (Das et al., 2013, 2014).

The best-characterized IMEX, which exploits the host encoded tyrosine recombinases for its lysogenic conversion is CTXΦ, a temperate filamentous bacteriophage that encodes the cholera toxin in *V. cholerae* (Waldor and Mekalanos, 1996). The CTXΦ recognizes the toxin co-regulated pilus (TCP), a type IV pilus encoded by the vibrio pathogenic island–I (VPI-1), and subsequently introduces its ~ 7.0 × 10^3^ nucleotides long circular single stranded genomic DNA (+ssDNA) into the *V. cholerae* cytoplasm. Once in the bacterial cytoplasm, ssDNA may convert to dsDNA, and start the rolling circle replication, or it can be recognized by the chromosomally-encoded tyrosine recombinases, that enable CTXΦ integration into the *dif* site of host chromosome. Tandemly integrated CTXΦ can also initiate the rolling circle replication from the chromosome and produce extrachromosomal ssDNA genome that may contribute in virion production (Davis and Waldor, 2000). Compared to other episomally replicative filamentous phages, the number of phage particles produced by the CTX-prophage is very low, even upon induction, resulting in one phage particle produced per 10–100 host cells (Davis et al., 2002). The other two well-characterized IMEXs that use the same recombinases for integration at the *dif1* site of *V. cholerae* are VGJΦ and TLCΦ (Campos et al., 2003b; Hassan et al., 2010; Das et al., 2011b). Presence of single or multiple copies of VGJΦ and TLCΦ were reported in toxigenic *V. cholerae* isolates. Unlike CTXΦ, the virion production of VGJΦ, and TLCΦ mainly relies on the episomal replication. Although the genomes of the two latter elements do not encode any toxins, they are nevertheless implicated in the host fitness and play important role in acquisition of the cholera-toxin-encoding genes and CTXΦ dissemination (Hassan et al., 2010; Das et al., 2011b; Midonet et al., in press).

In this review, I will update the mechanistic insights into filamentous vibriophage integration and cooperative interactions amongst IMGEs, which support the emergence of new pathogenic strains by contributing efficient acquisition and rapid dissemination of the cholera toxin genes in closely or distantly related bacterial strains.

## Xer recombination system in *Vibrio cholerae*

The native function of Xer recombination system is to ensure the stable maintenance of monomeric circular replicon (Blakely et al., 1993). In *V. cholerae*, the principal components of the Xer recombination system are (i) two recombinases, XerC and XerD; (ii) a DNA motor protein, FtsK; (iii) a 28-bp DNA sequence, *dif* (Val et al., 2008) (Table 1). The Xer proteins are members of the tyrosine recombinase family (Esposito and Scocca, 1997). Members of this family share two conserved motifs, containing four highly conserved residues (R1-H-R2-Y), a catalytic tyrosine, which acts as the nucleophile during cleavage of phosphodiester bond, two arginines and a histidine, which have been implicated both the cleavage and rejoining of DNA strands (Esposito and Scocca, 1997). The two positively charged arginines are thought to stabilize the pentavalent phosphate transition state, whereas the histidine may act as a general base catalyst (Esposito and Scocca, 1997; Sherratt and Wigley, 1998).

**Table 1:** **Key components of the Xer recombination system in *V. cholerae***.

**Gene**	**Gene ID in N16961 genome**	**Gene length (bp)**	**Protein**	**Protein length (aa)**	**Mol. weight (kD)**
*xerC*	*VC0128*	936	XerC	311	35.55
*xerD*	*VC2419*	909	XerD	302	34.56
*ftsK*	*VC1903*	2883	FtsK	960	105.89

**Figure 1:**
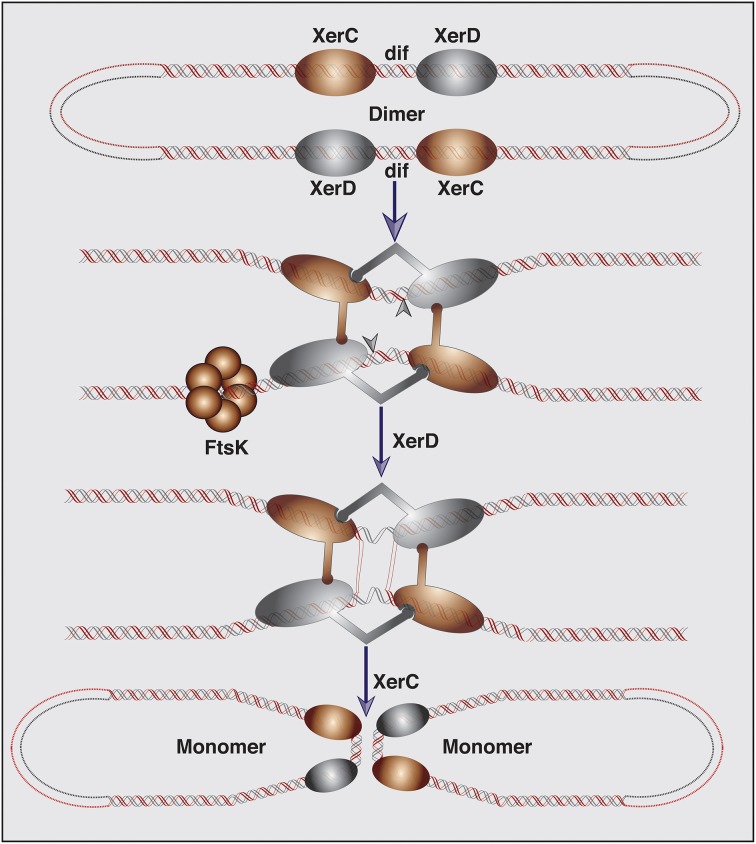
**Schematic representation of the XerC-XerD mediated chromosome dimer resolution**. Chromosome dimers are resolved by the XerC-XerD-mediated recombination at the *dif* site. DNA motor protein FtsK brings two *dif* sites together and induces the XerD catalytic activity required for the first pair of strand exchanges. Followed isomerization, XerC mediates the second pair of strand exchanges, and completes the recombination reaction.

During chromosome dimer resolution at the end of DNA replication, tetrameric XerC and XerD recombinase complex catalyzes successive strand exchanges between the two *dif* sites of a dimeric chromosome. The resolution is completed by a two-step transesterification reaction in which the OH- group of catalytic tyrosine residue of each recombinase is directly involved in the phosphodiester bond formation (Figure 1). In the first step of dimer resolution reaction, cleavage of a phosphodiester bond in DNA is introduced by the active enzyme, generating a covalently linked enzyme-DNA complex at the recombinase binding site and a free 5′-hydroxyl group at the end of the central region. Following the cleavage, the exposed sequence at the 5′-hydroxyl ends of the central region melt from their complementary strands and react with their recombinase-DNA phosphotyrosyl linked recombining partner. Subsequent joining between *dif* and *attP* strands requires complementary base pair interactions at the site of the phosphodiester bond formation (MacDonald et al., 2006; Das et al., 2010).

FtsK, a bifunctional protein essential for cell division and chromosome partitioning, induces the XerD function during chromosome dimer resolution (Recchia et al., 1999; Aussel et al., 2002; Demarre et al., 2013). The N-terminal part of FtsK forms a transmembrane domain and is directly linked to the forming septum. The cytoplasmic C-terminal domain is involved in the inter- and intracellular DNA transfer and activation of XerD recombinases (Bigot et al., 2006). Recent reports demonstrated that several IMGEs exploit the conserved Xer recombination system of bacteria to mediate their integration in the dimer resolution site of host chromosomes (Das et al., 2013). The integration mechanisms of such elements were studied in details in *V. cholerae* and revealed that the IMEXs present in the *dif* region of *V. cholerae* make use of three distinct integration mechanisms for lysogenic conversion (Das et al., 2010, 2011a,b, 2013; Midonet et al., in press).

## Lysogenic filamentous vibriophages exploit Xer recombinases

**Figure 2:**
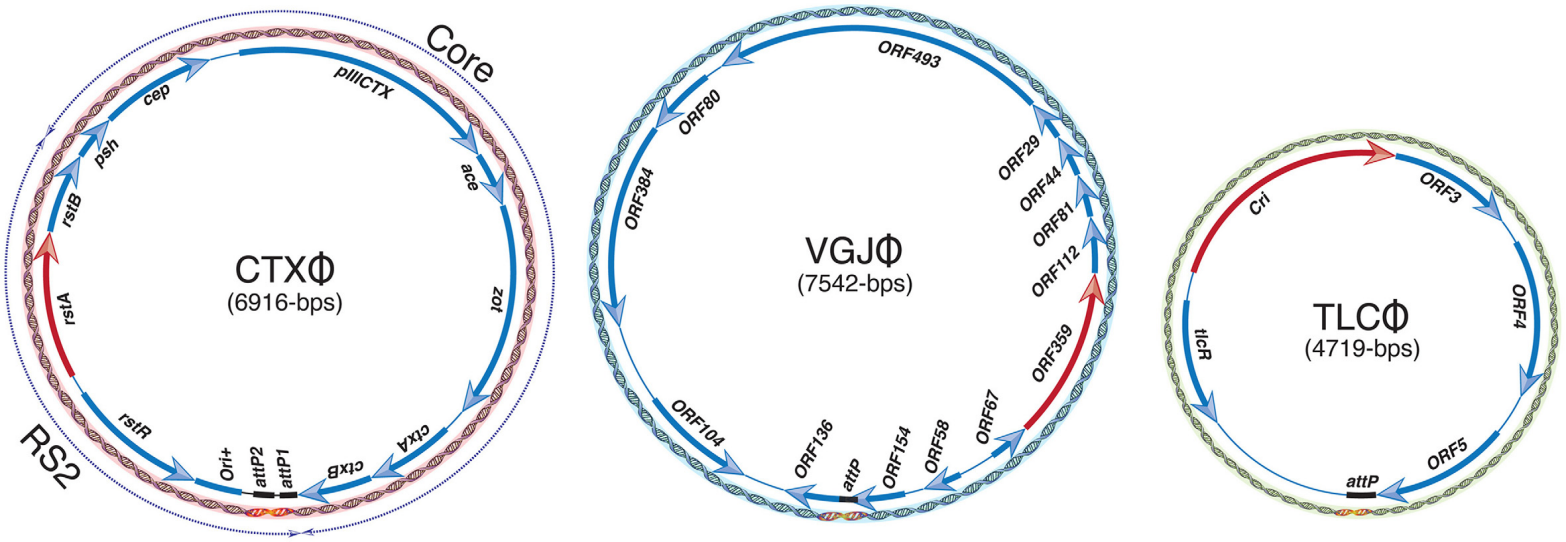
**Genomic organization of CTXΦ, VGJΦ, and TLCΦ phages**. A red arrow depicts gene essential for replication in each genome. A solid black line denotes attachment sequence, recognized by the Xer recombinases. Replicative form (RF) of each phage genome is presented as dsDNA. Arrows indicate the direction of transcription of each ORF. A thin solid line illustrates intergenic regions. Dashed arrows above the CTXΦ genome represent the core and RS2 modules.

Most of the characterized filamentous vibriophages are lysogenic and integrate at the *dif1* and/or *dif2* sites of *V. cholerae*. All reported filamentous vibriophages are equipped with an autonomously replicating genetic module, with or without toxin-encoding genes (Figure 2). Some of the filamentous vibriophages are directly responsible for *V. cholerae* pathogenesis, others contribute to toxin acquisition and host fitness (Waldor and Mekalanos, 1996; Hassan et al., 2010). Their genomes may or may not be equipped with genes required for virion production and can exploit the virion structural and assembly genes of other filamentous phages for genome packaging (Hassan et al., 2010). Although most of the filamentous vibriophages use host-encoded Xer machinery for their integration, their attachment sites (*attP*), and their genomic organization are fairly distinct (Figure 2). The best-studied CTXΦ genome is organized into two structurally and functionally distinct modules called repeat sequence 2 (RS2) and core (Figure 2). The RS2 module carries genetic traits essential for CTXΦ replication, maintenance of the ssDNA genome, and transcriptional regulation from phage originated P_RSTA_ promoter (Waldor et al., 1997). The replicating genome of CTXΦ is detrimental to *V. cholerae* growth, but is tolerated upon integration into the *dif* sites of either one or both chromosomes (Das et al., 2010; Faruque and Mekalanos, 2012). Compared to toxigenic strains, that contain a single or multiple integrated CTXΦ genomes, *V. cholerae* cells carrying a replicative form of CTXΦ grow slowly and rapidly lose the replicative phage both under the standard laboratory growth conditions and in rabbit gastrointestinal tract animal model (Faruque et al., 2001).

VGJΦ, a filamentous (+)ssDNA lysogenic bacteriophage, infects both clinical and environmental isolates of cholera pathogen and exploits the host-encoded Xer recombinases to integrate at the *dif1* site in the large chromosome of *V. cholerae* (Campos et al., 2003b, 2010; Das et al., 2011b). In contrast to complex attachment site of CTXΦ that contains two *dif*-like *attP* sites in an inverted orientation, the 7.5-kb genome of VGJΦ harbors a single *dif*-like 29-bp attachment sequence, called *attP*^*VGJ*^ and integrates specifically into the *dif1* site.

TLCΦ, a satellite temperate phage with an autonomous replication module but lacking the morphogenesis and structural virion-encoding genes, is often present in the *dif1* region of toxigenic *V. cholerae* isolates (Rubin et al., 1998). Recent reports demonstrated that, like CTXΦ, and VGJΦ, TLCΦ also relies on the host-encoded Xer machinery for establishing lysogeny (Hassan et al., 2010; Midonet et al., in press). Interestingly, although both the XerC and XerD recombinases are essential for integration of IMEXs, none of the reported IMEXs rely on FtsK for recombination.

Different vibriophages have different *attP* structures and therefore rely on to a certain extent of different mechanisms of interaction with the Xer recombinases and integration (Das et al., 2013).

### Mechanistic insights into lysogenic conversion of vibriophages

**Figure 3:**
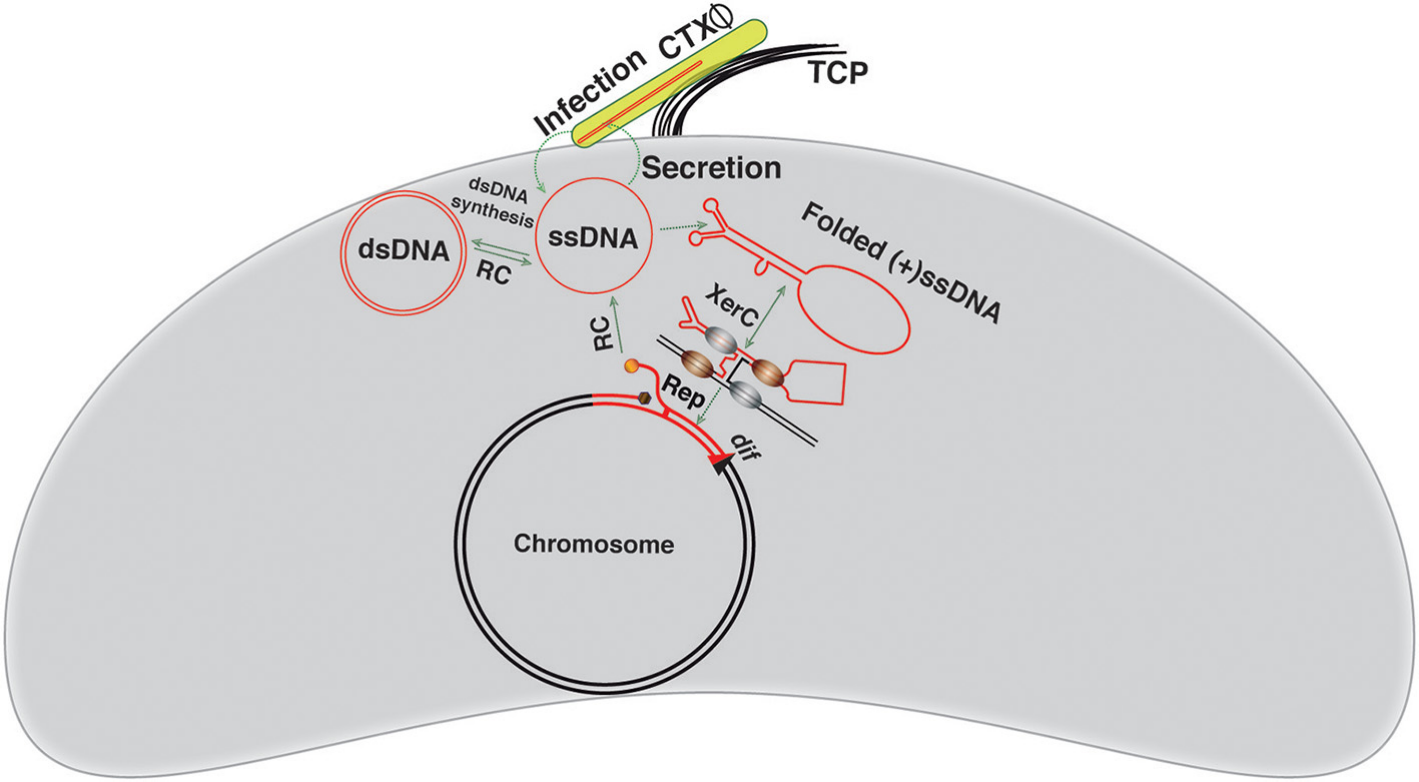
**Visual depiction of the CTXΦ integration and replication**. The CTXΦ virion recognizes the cognate *V. cholerae* cell surface receptor TCP and delivers its ssDNA genome into the *V. cholerae* cytoplasm. The ssDNA genome of CTXΦ may be converted into dsDNA or directly integrated into a chromosomal *dif* site using the host-encoded XerC-XerD recombinases. XerC mediates first pair of strand exchanges. The host DNA replication, possibly, resolves the resulting Holiday junction. The red line depicts the phage genome, while a black line shows bacterial genome. Newly generated *dif* site is represented by a triangle.

**Figure 4:**
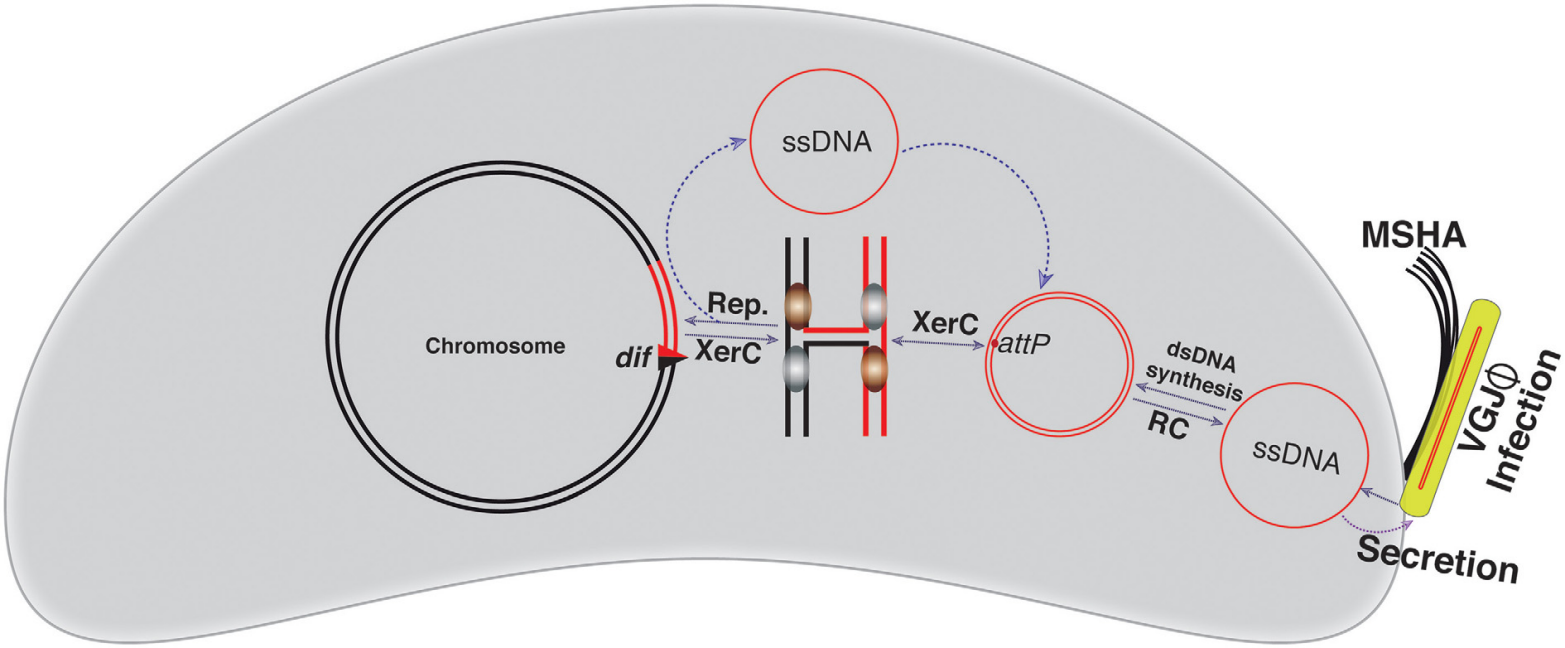
**Schematic representation of the VGJΦ integration and replication**. VGJΦ uses mannose-sensitive hemagglutinin A (MSHA) to as a primary host receptor and delivers its ssDNA genome into the host cytoplasm. The ssDNA genome of VGJΦ is first converted into dsDNA and for this it contains a *dif* like DNA sequence, *attP*^*VGJ*^. XerC mediates the first pair of strand exchanges and the host DNA replication resolves the resulting Holiday junction. The VGJΦ integration is reversible. Excision follows the same sequence of strand exchanges as described for integration. The red line depicts the phage genome while a black line depicts bacterial genome. Newly generated *dif* site is represented by a triangle.

**Figure 5:**
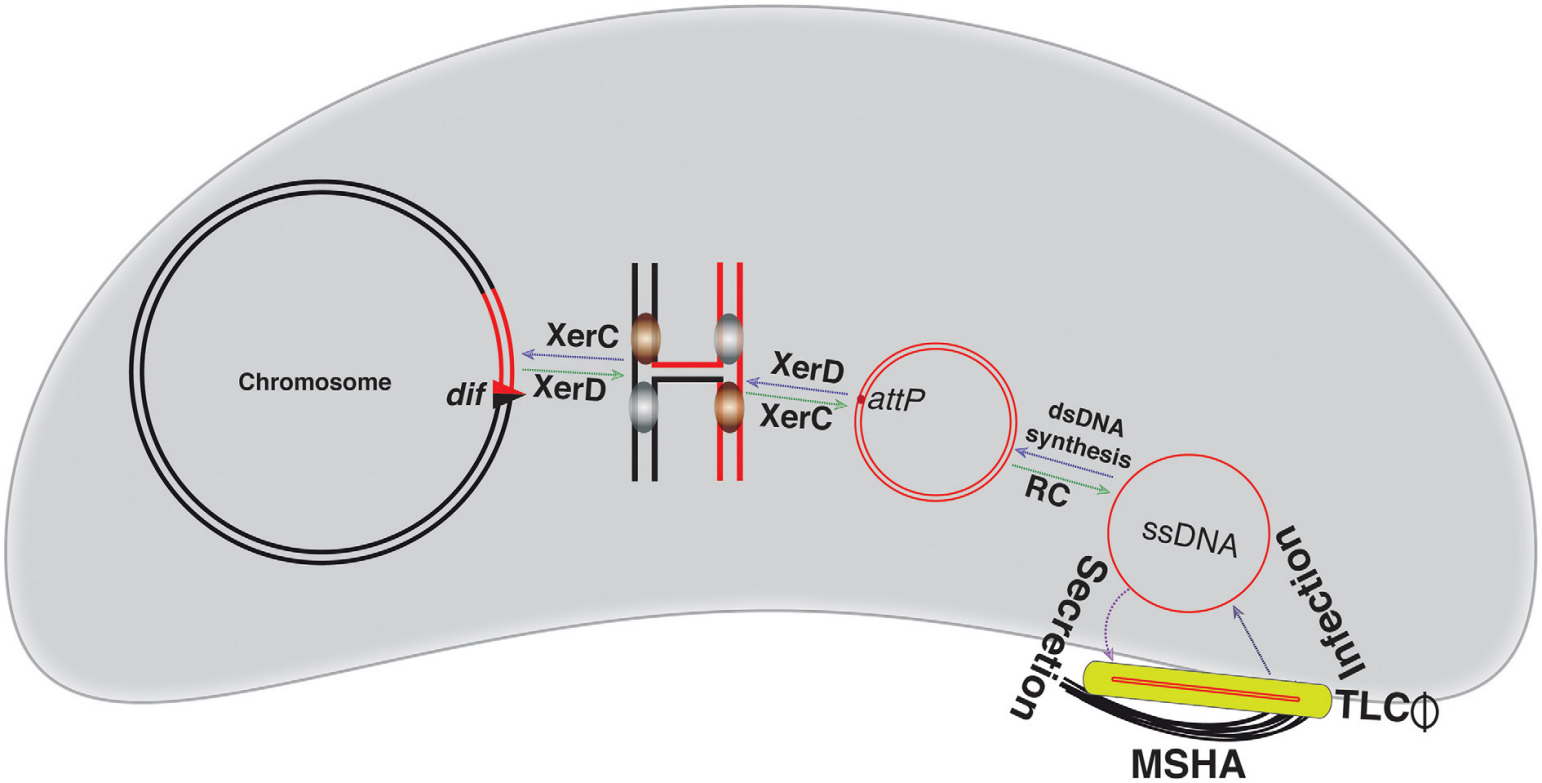
**Illustration of the TLCΦ integration and replication**. The TLCΦ satellite prophage uses morphogenesis proteins of other filamentous phages, like fs2 for assembly. It uses the mannose-sensitive hemagglutinin A (MSHA) as the primary host receptor and delivers its ssDNA genome into host cytoplasm. The ssDNA genome of the TLCΦ is first converted into dsDNA. The *attP*^*TLC*^ in the dsDNA replicative genome of TLCΦ is recognized by the XerC-XerD recombinases. XerD mediates the first pair of strand exchanges and generates a Holiday junction. After isomerization, XerC mediates second pair of strand exchanges and enables the TLCΦ integration. TLCΦ integration, which is reversible. Excision follows the same sequence of strand exchanges as described for integration. The blue and green arrows indicate integration and excision pathways, respectively. The red line depicts phage genome, while a black line represents bacterial genome. Newly generated *dif* site is symbolized by a triangle.

Most lysogenic filamentous vibriophages are IMEXs that depend on the host-encoded Xer recombinases for their lysogeny. The attachment site in the phage genome mimics the native chromosomal Xer binding sites and exploits Xer recombinases to catalyze site-specific recombination and enable phage integration. Based on the *attP* structure and integration mechanisms, vibriophages can be categorized into three classes (Table 2). Although the constituents of the integration reaction are very similar, integration is achieved by three distinct mechanisms, where catalytic activity of recombinases, order of strand exchanges and the resolution of reaction intermediates into end products are different (Figures 3–**5**). In this review, I will concentrate on CTXΦ, VGJΦ, and TLCΦ lysogenic conversions to describe the most updated integration mechanisms of these phages studied in *V. cholerae* and related bacterial cells.

**Table 2:** **Different components and strategies used by three different filamentous Vibriophages for their integration at *dif* sites of *V. cholerae* chromosome**.

**Class**	**CTXΦ**	**VGJΦ**	**TLCΦ**
Integration site in chromosome	*dif1/dif2*	*dif1*	*dif1*
Phage attachment site	*attp(+) (~150 nt)*	*attp^VGJ^ (29-bp)*	*attp^TLC^ (28-bp)*
Integrative phage DNA	(+)ssDNA	dsDNA	dsDNA
Essential components for integration	XerC, XerD, *attp(+)*, *dif1/dif2*	XerC, XerD, *attp*^*VGJ*^, *dif1*	XerC, XerD, *att*^*TLC*^, *dif1*
First pair of strand exchanges is mediated by	XerC	XerC	XerD
Integration is completed by	Host DNA replication	Host DNA replication	XerC
Integration event	Irreversible	Reversible	Reversible
Excision initiated by	–	XerC	XerD
Excision completed by	–	Host DNA replication	XerC
Prophage to phage production depends on	Rolling circle replication	Excision	Excision
Related members	RS1, YpfΦ, CUS-1Φ	VEJΦ, VSKK, VSK, fs2, Vf12, VfO4K68	ΦLf, XfΦf1, Cf1c, GGI

### Mechanistic insights into CTXΦ integration

CTX-prophages are ubiquitously present in the chromosomes of all epidemics *V. cholerae* isolates (Mekalanos et al., 1983; Mekalanos, 1985; Vanden Broeck et al., 2007). Phage to prophage conversion is rapid and efficient process achieved through site-specific recombination between *attP*(+) of CTXΦ and *dif* sites of *V. cholerae* (Val et al., 2005) that results in a stable integration. The functional *attP*(+) structure is formed by intra-strand base pairing interactions between two inversely oriented *attP* sequences located between the *ctxB* and *rstR* genes in the circular CTXΦ genome (Figure 2). In a replicating form (RF) of the genome, *attP1* consists of XerC and XerD binding sites separated by a 12 bp central region. The *attP2* is consists of 11-bp XerC and XerD binding sites and a 5–7 bp central region. Neither *attP1* nor *attP2* are suitable for Xer-mediated integration due to extended central region or presence of incompatible bases in the central region, respectively (Das et al., 2010). The specificity and compatibility of CTXΦ integration is solely determined by the homology of bases next to the XerC-binding site between the overlapping (or corresponding) regions of *attP* and *dif* sequence (Das et al., 2010). A 90-bp DNA fragment separates the *attP1* and *attP2* of CTXΦ. The CTXΦ integration sites in *V. cholerae* chromosomes (*dif1* and *dif2*) each consist of 28 bp DNA sequence. In *dif1*, a 6 bp central region, at the border of which the strand exchange occurs, separates the 11 bp binding sites of XerC and XerD. The chromosomally-encoded Xer recombinases can recognize both the double stranded chromosomal *dif* site and folded single stranded *attP*(+) of CTXΦ and catalyze recombination to facilitate the phage ssDNA integration.

The CTXΦ ssDNA integration takes place within a DNA-protein complex, where DNA strands are cleaved and rejoined sequentially by XerC recombinase through transient nucleoprotein covalent intermediates. The catalytic tyrosine residue of Xer recombinases functions as a nucleophile and mediates the cleavage of a phosphodiester bond between the last base of the recombinase-binding site and the first base of central region. The key components within the nucleoprotein complex are XerC, XerD, *attP*(+), and *dif* sequences. Although the presence of both recombinases are essential for recombination, successful integration of the CTXΦ ssDNA needs only the catalytic activity of XerC (Val et al., 2005). At the onset of recombination reaction, XerC introduces a cut of the phosphodiester bond between the last base of XerC binding site and the first base of the central region, of *dif1/dif2*, and *attP*(+) (Figure 3). On one side, a XerC-DNA complex is formed through a covalent phosphotyrosyl bond. On the other side, the DNA strand with a free 5′-OH end melts away from the central region of the reaction complex, migrates to the central region of the partner strand and forms complementary base pairing interactions to stabilize the exchanged strands and form of a phosphodiester bond between the deoxyriboses of two adjacent nucleotides. This step is the determining factor for phage integration specificity (Das et al., 2010). The resulting strand exchanges generate a Holiday Junction (HJ) intermediate. Host DNA replication, possibly, resolves the resulting, transient HJ intermediate and accomplishes the CTXΦ ssDNA integration into the *V. cholerae* chromosome (Figure 3). The absence of any homology between central region bases at the XerD-side of *attP*(+) and *dif1/dif2* impedes any XerD mediated strand exchange. A recent report revealed that the host-encoded DNA repair protein, endonuclease III, a product of *nth* gene with N-glycosylase and AP-lyase functions, facilitates the CTXΦ genome integration, possibly by stabilizing the transient HJ intermediate (Bischerour et al., 2012). Each individual step in the CTXΦ genome integration pathway is in principle reversible, but the prophage excision has never been detected under the standard laboratory conditions. This is due to the loss of a functional folded *attP*(+) structure in the double-stranded form of prophage (Val et al., 2005), in which the intra- strand base pairing between *attP1* and *attP2* is excluded. In the prophage genome, both *attP* sequences retained the XerC and XerD binding sites, but the central region of both sites, where the strand exchange occurs during integration, are incompatible with the Xer reaction and make the CTXΦ integration irreversible (Das et al., 2010).

### Mechanistic insights into VGJΦ integration

VGJΦ, a filamentous (+)ssDNA lysogenic bacteriophage, infects both clinical and environmental isolates of cholera pathogen and exploits the host-encoded Xer recombinases to integrate at the *dif1*site in the large chromosome of cholera pathogen (Campos et al., 2003b; Das et al., 2011b). In contrast to CTXΦ, the 7.5 kb genome of VGJΦ harbors a single *dif* like 29-bp DNA sequence, called *attP*^*VGJ*^ (Figure 2). Presence of the single functional *dif* like site allows integration of VGJΦ genome into the *V. cholerae* chromosome as dsDNA. Like CTXΦ, integration of VGJΦ also relies on the catalytic activity of XerC (Figure 4). Due to an absence of the sequence homology in the central region adjacent to the XerD-binding site between the *attP*^*VGJ*^ and *dif1* site of *V. cholerae*, XerD catalytic activities are not been used either to generate HJ or to resolve the HJ during integration. The XerC generated HJ junction is resolved either by the host DNA replication or other DNA repair proteins and assist VGJΦ integration (Figure 4). After integration, two compatible Xer recombination sites (*attL* and *attR*) flank the VGJ-prophage. The 7.5-kb distance between the two Xer binding sites does not impede assembly of the recombination complex and excision of the VGJΦ prophage. Like integration, excision of VGJΦ also depends on the XerC-mediated strand exchanges (Figure 4). *In vitro* recombination reactions between synthetic *attP*^*VGJ*^ and *dif1* substrates confirmed that only XerC and XerD recombinases are sufficient for DNA rearrangement (Das et al., 2011b).

### Mechanistic insights into TLCΦ integration

TLCΦ, a satellite temperate phage with an autonomous replication module, is mostly present in the *dif1* region of toxigenic *V. cholerae* isolates. Recent reports demonstrated that like CTXΦ, and VGJΦ, TLCΦ also relies on host encoded Xer machinery for its lysogeny (Hassan et al., 2010; Midonet et al., in press). In sharp contrast to the first two elements, the XerD binding site of TLCΦ is degenerated (Das et al., 2013; Midonet et al., in press). The XerC binding site and central region of *attP*^*TLC*^ are almost identical to *dif1* of *V. cholerae*, but XerD binding sequence has very little similarity (Midonet et al., in press). Despite the absence of bona fide XerD binding site in *attP*^*TLC*^, TLCΦ integration is strictly related to XerD catalytic activity (Midonet et al., in press). The integration mechanism of TLCΦ is unique compared to the integration strategy adopted by CTXΦ, and VGJΦ (Table 2).

Like CTXΦ and VGJΦ, TLCΦ integration also needs both XerC and XerD recombinases. During TLCΦ integration, XerC and XerD form a hetero-tetrameric complex with *dif1* and *attP*^*TLC*^, within which XerD exchanges first pair of DNA strands and form the HJ. The resulting HJ proceed to DNA isomerization and is subsequently resolved by the XerC-mediated second strand exchanges (Figure 5). Tyrosine recombinases-mediated reactions are reversible, and therefore the TLCΦ integration is also reversible. Like integration excision is also depends on XerD catalytic activity (Figure 5). The action of both recombinases and the sequence of strand-exchanges stages in both integration and excision are very similar to chromosome dimer resolution, however the TLCΦ integration occurs without direct participation of DNA motor protein FtsK. Since the recombination reaction between *dif1* and *attP*^*TLC*^ is not reconstituted in defined *in vitro* reactions, it is yet not clear whether only the 28-bp *attP*^*TLC*^ is sufficient for Xer reaction or whether the TLCΦ needs extended *attP*^*TLC*^ region and support from additional host proteins for efficient integration. Similarly, the mechanistic insights into TLCΦ excision in defined reaction conditions are yet to be explored.

### Cooperative interactions among filamentous vibriophages

Metagenomic studies revealed several IMEXs integrated at one or both chromosomes of cholera pathogenic strains (Chun et al., 2009). The co-occurrence of multiple IMEXs in the genome of cholera pathogens is not simply a coincidence. Recent reports demonstrated that there are remarkable cooperative interactions between closely or distantly located IMEXs in cholera pathogen (Taylor et al., 1986; Hassan et al., 2010; Das et al., 2011b). Interactions between IMEXs happen at different levels, including the host recognition, receptor binding and DNA entry into the cytoplasm, chromosomal integration, and virion production (Waldor and Mekalanos, 1996; Hassan et al., 2010). The best example of this interaction is acquisition of cholera toxin. The first step of CTXΦ infection in *V. cholerae* is to recognize its host receptor. TCP, the cell surface receptor of *V. cholerae* for CTXΦ, is encoded by VPI-1, a ~41-kb mobile genomic island. The same genomic island also helps cholera toxin production by providing transcriptional inducer ToxT, which specifically binds to the “TATTA” repeat, invariably present upstream of the *ctxAB* operon.

Cooperative interactions between VGJΦ and CTXΦ have been reported very recently (Campos et al., 2003a; Das et al., 2011b). CTXΦ integration is irreversible and phage production depends on the presence of tandem copies of prophages or related RS1 elements (Waldor et al., 1997; Val et al., 2005). A recent report demonstrated that VGJΦ could help CTXΦ excision and its dissemination to the *V. cholerae* strains devoid of TCP island. When the CTXΦ prophage is inserted between the attachment site of VGJΦ and the *dif1* of *V. cholerae*, a hybrid VGJΦ-CTXΦ is often detected (Das et al., 2011b). The hybrid phage genome is packed within the VGJΦ coat proteins and infects *V. cholerae* cells expressing mannose-sensitive hemagglutinin (MSHA) pilus, a type IV pilus present on the cell surface whose structural pilin subunit is encoded by the *mshA* gene. MSHA pilus is ubiquitous and constitutively expressed in all *V. cholerae* serotypes and thus, host range of the hybrid phage containing the CTXΦ genome encapsulated in the VGJΦ-encoded virion is not restricted to the cells that expressed TCP (Fullner and Mekalanos, 1999). Similarly, TLCΦ also helps the CTXΦ excision if both elements are present in tandem in the *V. cholerae* chromosome (Midonet et al., in press). Since the TLC prophage is present in most toxigenic strains where a single or multiple copies of the CTXΦ genome are integrated at the chromosomal *dif1* site, the TLCΦ is more significant for the CTXΦ excision than is the VGJΦ-prophage. Other than TLCΦ and VGJΦ elements, several other IMGEs could help CTXΦ replication as well as its interaction with the *V. cholerae* in natural environment by providing cell surface receptor. They can also help increase the amount of cholera toxin production by up-regulating *ctxAB* operon, protein required for the hybrid virion production and dissemination in the environment.

## Conclusions

In this review, several fundamental questions related to IMEXs integration have been addressed, such as contribution of these elements to continuous evolution of the cholera pathogenic strains. Although hypothesis for the adaptive advantage of lysogenic conversion of filamentous vibriophages was proposed, many important questions are still open and need to be addressed in order to improve understanding of the *V. cholerae* biology and cholera management (Box 1). Pangenomic studies revealed that IMEXs are not limited only to clinical isolates but are also widely distributed in the environmental *V. cholerae* isolates that are closely or distantly related to pathogenic strains. Much emphasis has been put on the biology of those IMEXs that are mostly present in the clinical isolates. Our knowledge on IMEXs present in the environmental isolates is limited. It is widely accepted that the environmental *V. cholerae* strains are ubiquitously distributed in aquatic environment and could serve as a reservoir of toxin-encoding genes as well as other fitness factors for clinical strains. Comprehensive studies of IMEXs present both in clinical and environmental isolates of *V. cholerae* and related pathogenic strains are required in order to understand the microevolution of the species pertinent to epidemiology. On the other hand, better mechanistic and structural knowledge of IMEXs will help to develop therapeutic agents and limit the emergence of the new cholera pathogenic strains and other strains that may pose a serious threat to human beings as well as animals.

Box 1 **Outstanding questions.**

❖ How is the CTXΦ integration efficiency modulated? Does TLCΦ modulate efficiency of CTXΦ integration into the *V. cholerae* chromosomes?
❖How is the CTXΦ virion produced from toxigenic *V. cholerae* cells, in the case when it harbors only a single copy of the prophage in either chromosome?
❖Does the TLCΦ contribute to the CTXΦ rolling circle replication?
❖Is there any relation between the LexA regulon and the CTXΦ integration? Does ssDNA of CTXΦ induced the SOS response in the host cell?
❖How does the TLCΦ genome integrate in the chromosome of classical *V. cholerae* strains, where both chromosomes contained an *attP*^*TLC*^ incompatible *dif2* sequence?
❖Are there any accessory proteins other than Xer recombinases that may help the TLCΦ genome integration into the *V. cholerae* chromosome?
❖What are the host factors implicated in the CTXΦ, VGJΦ, and TLCΦ replication?

### Conflict of interest statement

The Guest Associate Editor, Jasna Rakonjac, declares that, despite having collaborated on the same research topic as author Bhabatosh Das, the review process was handled objectively and no conflict of interest exists.
